# Activation of the Prefrontal Cortex While Performing a Task at Preferred Slow Pace and Metronome Slow Pace: A Functional Near-Infrared Spectroscopy Study

**DOI:** 10.1155/2014/269120

**Published:** 2014-11-10

**Authors:** Kaori Shimoda, Yoshiya Moriguchi, Kenji Tsuchiya, Shiori Katsuyama, Fusae Tozato

**Affiliations:** Department of Rehabilitation, Gunma University Graduate School of Health Sciences, 3-39-22 Showa, Maebashi, Gunma 371-8514, Japan

## Abstract

Individuals have a preferred pace at which they perform voluntary repetitive movements. Previous studies have reported that greater activation of the prefrontal cortex was observed during self-initiated movements than during externally triggered movements. The purpose of the present study is to compare the activation of the prefrontal cortex induced when the subjects performed a peg-board task at their preferred slow pace (PSP, the self-initiated condition) with that induced when they performed the same task at metronome slow pace (MSP, the externally triggered condition) using functional near-infrared spectroscopy. Healthy subjects performed the task while sitting in a chair. By assessing the activated channels individually, we confirmed that all of the prefrontal regions of interest were activated by both tasks. In the second-level analyses, we found that the activation detected in the frontopolar cortex (FPPFC; Brodmann area 10) was higher during the PSP task than during the MSP task. The FPPFC is known to be at the top of prefrontal hierarchy, and specifically involved in evaluating self-generated information. In addition, the FPPFC plays a role in coordinating lateral prefrontal cortex. In the present study, the subjects evaluated and managed the internally generated PSP by coordinating the activity of other lower level prefrontal regions.

## 1. Introduction

Individuals are known to have a preferred pace when performing voluntary repetitive movements [1], and this has been shown to differ among individuals and is moderately stable [2–4].

The contribution of different brain areas to the internal versus external guidance of movement remains controversial. Several neuroimaging studies reported that activation of the supplementary motor area (SMA) was greater during self-initiated movements [5–8]. However, the cerebellum showed to be strongly associated with externally triggered movements [9].

Jahanshahi et al. compared self-initiated and externally triggered finger extensions at a mean frequency of once every 3 s using positron emission tomography (PET). The self-initiated condition involved self-generated “when to do” decisions regarding the time to initiate movements. Activation of the right dorsolateral prefrontal cortex (Brodmann areas 9, 46, and 10) was greater during self-initiated than externally triggered movements [10]. In a subsequent study, the subjects performed finger extensions at random intervals between 2 and 7 s, and the results obtained revealed that the bilateral dorsolateral prefrontal cortex was activated during the self-initiated task [8]. However, the subjects that participated in these experiments performed the task while lying supine with their eyes closed in a darkened room. Therefore, this experimental environment did not reflect the natural conditions of daily life.

The number of neuroimaging studies using functional near-infrared spectroscopy (fNIRS) has increased recently. The fNIRS technique was first described in 1977 [11]. fNIRS utilizes noninvasive light in the wavelength range 700–1000 nm to transilluminate cortical tissues [12]. fNIRS is an effective and indirect optical neuroimaging method that monitors hemodynamic responses to brain activation on the basis of neurovascular coupling, and specific parameters of the response monitored with fNIRS comprise changes in oxygenated and deoxygenated hemoglobin at specific loci across the cortical surfaces that are evoked following stimulation [13]. The advantage of fNIRS technology is that the equipment used is portable, discreet, and relatively robust to motion artefacts, which has enabled human infant and adult cortical correlates of realistic everyday tasks to be delineated [13, 14]. Several studies have used fNIRS to examine everyday motor behaviors such as talking [15], walking (on the treadmill) [16], and peeling an apple [17].

We previously compared activation of the prefrontal cortex when the subjects performed a task at preferred pace and metronome pace using fNIRS. In our past and the present studies, preferred pace refers to a self-initiated condition, while metronome pace indicates the externally triggered condition defined by Jahanshahi et al. [10]. Our subjects performed finger tapping with the left (nondominant) hand while sitting in a chair. We demonstrated that activation of the whole prefrontal cortex was greater during the preferred pace task than during the metronome pace task [18]. However, a limitation of the study was that we could not get the information about fNIRS locations of optodes correctly. Therefore, in the present study, we again compared activation of the prefrontal cortex at each region of interest when the subjects perform a task at preferred pace or metronome pace using fNIRS. The selected speed was slow pace. We speculate that when the subjects perform the metronome pace task, slow pace would be the easiest to synchronize with the external cue because there should be adequate time to prepare for the next cue.

## 2. Materials and Methods

### 2.1. The Subjects

Twenty-two healthy university students (13 males; 9 females: average age = 20.4 years; SD = 1.2; range: 19–23 years) participated in the present study. All the subjects were confirmed to be right-handed by the Edinburgh Inventory [19]. Written informed consent was obtained from each subject. The present study was approved by the Institutional Review Board of Gunma University.

### 2.2. Task

**Figure 1:**
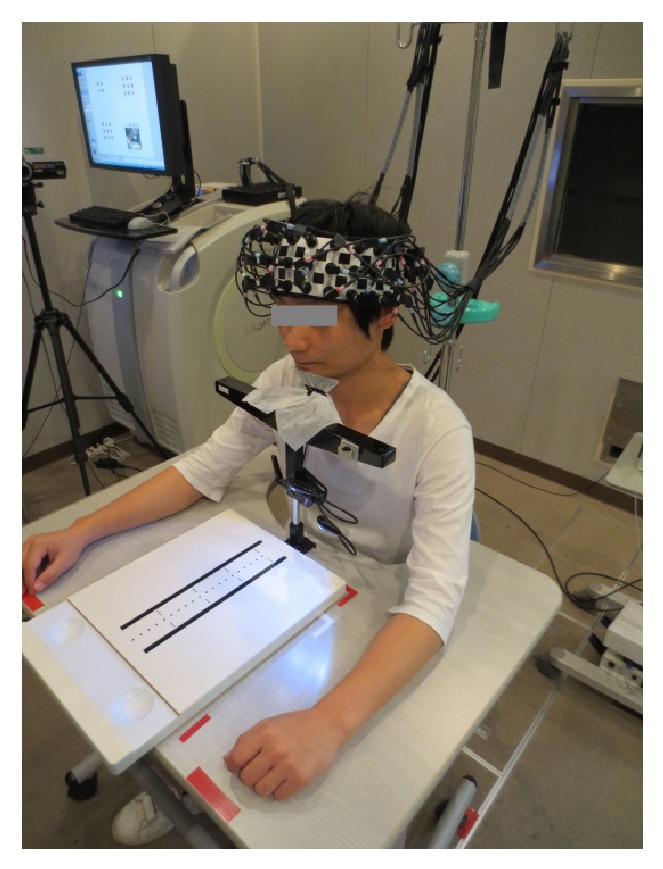
Experimental environment. The subjects performed the pegboard task at preferred slow pace (PSP) and metronome slow pace (MSP) during functional near-infrared spectroscopy recording.

The subjects sat in a chair with a backrest in a quiet, dimly lit, and electrically shielded room. They rested their jaws on a stand (T. K. K. 930a, Takei Scientific Instruments Co., Ltd., Niigata, Japan) to prevent the effects of head tilt on fNIRS recording. The subjects performed the pegboard test, which is a subtest of the manual function test (SOT-5000, SAKAI Medical Co., Ltd., Tokyo, Japan) that is frequently used to assess patients with cerebrovascular diseases [20, 21] (Figure 1). The pegboard test assesses integrated function in the arm including finger dexterity using the pegboard. The patient transfers pegs from a saucer to the board one at a time and as quickly as possible, and the total number of pegs correctly transferred within 30 s is counted [22].

**Figure 2:**
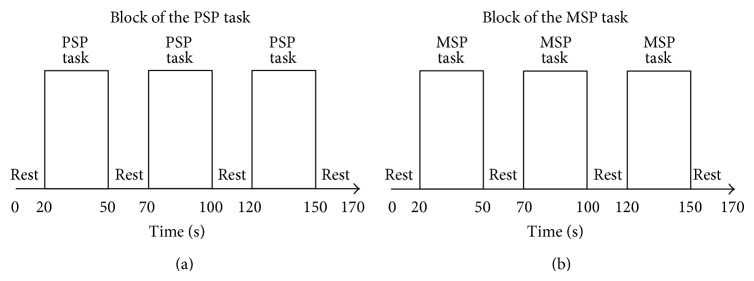
The task paradigm. That was a periodic block design involving rest (20 s) and task (30 s) blocks. All the subjects performed three sets of PSP task and rest blocks, followed by three sets of MSP task and rest blocks.

In the present study, the subjects performed the pegboard test with their right hands. Two different paces were examined: preferred slow pace (PSP) and metronome slow pace (MSP). In the PSP task, the subjects were instructed to perform the task like walking as slowly as possible. The subjects had to decide when to initiate movements and maintain their PSP. The subjects performed the MSP task at a customized metronomically regulated pace based on their individual PSP. During the rest period in both tasks, the subjects placed their right hand on the desk and stared at the mark. During the rest periods in the MSP task specifically, the subjects rested while listening to the metronomic tones from a digital quartz metronome (ME-55, YAMAHA Corp., Shizuoka, Japan). Prior to the fNIRS recording, all of the subjects were trained to perform the two tasks smoothly. The experiment had a periodic block design involving rest (20 s) and task (30 s) blocks. As for the block order, three sets of PSP task and rest blocks were performed, followed by three sets of MSP task and rest blocks (Figure 2). A fixed order was used because the rate at which the metronomic tones were presented had to match the rate of the subject's movements in the PSP task. Therefore, the experiment task was designed so that there would be no difference in the number of pegs moved between the PSP and MSP tasks.

### 2.3. fNIRS Recording

We employed an fNIRS system (LABNIRS, Shimadzu Corp., Kyoto, Japan) to detect cortical activation. This system used 3 wavelengths (780, 805, and 830 nm) of continuous near-infrared light and was based on the modified Beer-Lambert law [23]. We used 27 optodes that consisted of 14 light emitters and 13 light detectors in order to obtain 42-channel measurements. The interoptode distance was 3.0 cm. Optodes on the skull covered the frontal area. The localization was based on lower center probe, which was anchored at Fpz according to international 10–20 system and was located at the midpoint between channels numbers 38 and 39. The sampling time was set to 0.045 s. The optodes measured relative oxygenated hemoglobin (oxy-Hb), deoxygenated hemoglobin (deoxy-Hb), and total hemoglobin (total-Hb) signal changes in the prefrontal cortex.

**Figure 3:**
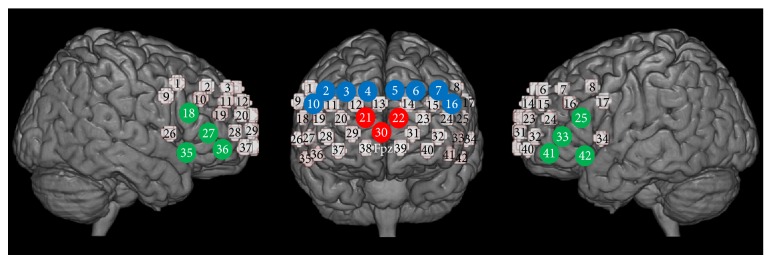
Localization of regions of interest. The numbers on small spheres on the brain map indicate the 42 channels. The channel localization was based on lower central probe, which was anchored at Fpz according to international 10–20 system and was located at the midpoint between channels numbers 38 and 39. The red channels (numbers 21, 22, and 30) were located in the FPPFC (BA 10), the blue channels (numbers 2, 3, 4, and 10 on the right and numbers 5, 6, 7, and 16 on the left) were located in the DLPFC (BA 9 and 46), and the green channels (numbers 18, 27, 35, and 36 on the right and numbers 25, 33, 41, and 42 on the left) were located in the VLPFC (BA 44, 45, and 47).

After the fNIRS experiment, we marked locations of optodes with 3D digitizer (FASTRAK, Polhemus, Colchester, VT, USA). Then, we identified the midpoints between neighboring emitters and detectors on the skull surface and used these as fNIRS channel loci. We used a probabilistic registration method to localize each channel on a standard cortical atlas. The virtual registration software [24] included in the NIRS-SPM [25] was used to perform this task. This enabled us to localize the channels' coordinates on the Montreal Neurological Institute (MNI) standard template [26] and probabilistically estimate structural labels (Brodmann areas (BA)) for the channels' coordinates. The Talairach Daemon [27] and MRIcro [28] included in NIRS-SPM [25] were also used to determine the labels for each coordinate. As a result, we set five regions of interest (ROIs), each of which contained 3 or 4 channels. Channels numbers 21, 22, and 30 were localized in the frontopolar cortex (FPPFC; BA 10; 100% probability, based on Talairach Daemon [27] and MRIcro [28]). Channels numbers 2, 3, 4, and 10 were determined to fall within the right dorsolateral prefrontal cortex (DLPFC; BA 9 and 46), and channels numbers 5, 6, 7, and 16 were considered to be located in the left DLPFC (more than 69.1% probability, based on Talairach Daemon [27]). Channels numbers 18, 27, 35, and 36 were located in the right ventrolateral prefrontal cortex (VLPFC; BA 44, 45, and 47), and channels numbers 25, 33, 41, and 42 were considered to fall within the left VLPFC (more than 44.6% probability, based on Talairach Daemon [27]) (Figure 3).

### 2.4. Analysis

We used the oxy-Hb in further statistical analyses because it was previously shown to be the most sensitive indicator of changes in fNIRS measurements [29]. We excluded data containing major artifacts due to insufficient optode contact.

#### 2.4.1. Activated Channels 

*(1) Activated Channels of Individual Analyses.* In the first-level individual analyses, the oxy-Hb time-series signals for the entire experimental session were subjected to a within-subject general linear model (GLM) using LABNIRS to calculate the variation in activation between the PSP task and rest and between the MSP task and rest. In this GLM analysis, hypothetical hemodynamic curves (convolved with the Gaussian hemodynamic response), in which the data for each task were compared with the baseline rest, were produced, with covariation of discrete cosine transformation terms that function as a high-pass filter. The variation in task-related activity (PSP versus rest and MSP versus rest) was evaluated using channel-by-channel *t* tests (*P* < 0.0001 after Bonferroni correction). The results of the GLM analyses were used to select positively activated channels (i.e., channels of interest (COIs)) whose activity passed the statistical threshold in the subjectwise analyses. Hence, it was possible for each subject to have different number of COIs for the PSP and MSP tasks.

To confirm whether the number of selected channels differed between the PSP and MSP tasks, we compared the mean number of activated channels within each ROI (for all the subjects) between the two tasks using paired *t* tests. IBM SPSS Statistics (ver. 22) was used for these analyses, and the level of significance was set at *P* < 0.05.

*(2) Activated Channels of Group Analyses*. The fNIRS data were also analyzed using NIRS-SPM [25] to identify the activation of all the subjects during these tasks. GLM was employed and the level of significance was set at *P* < 0.05. We used this result for only making figures.

#### 2.4.2. The Integral of the Oxy-Hb Signals

A baseline correlation was performed on LABNIRS prior to the analyses. When average intensity value was taken at an optional data range area in the experiment, the linear expression with 0 inclination, which passed this point, was taken as the background. We set the optional data range area with points of 0 s and 5.0 s in the rest period just before the initiation of the task period.

Before the analyses, we extracted the integral of the oxy-Hb signals for each 30 s task period separately and subjected the fNIRS data in each ROI to two-factor repeated measures analysis of variance (ANOVA), with the type of task (PSP or MSP) and three consecutive time bins as within-subject factors. No task × time-bin interactions were detected [*F* (2, 42) = 0.086, *P* = 0.92 for the FPPFC;* F* (2, 42) = 0.24, *P* = 0.79 for the right DLPFC;* F* (2, 42) = 0.16, *P* = 0.85 for the left DLPFC;* F* (1.42, 29.9) = 1.15, *P* = 0.31 for the right VLPFC; and* F* (2, 42) = 0.50, *P* = 0.61 for the left VLPFC]. Our results showed that the activity induced by the two different tasks changed in parallel across the three time bins. Thus, even if learning and repetition had had some effect on the time course of activation in each task, it did not affect the differences in activity between the PSP and MSP tasks; therefore, any differences in activity between the two tasks were purely task dependent. This enabled us to use the oxy-Hb signals that had been averaged across the three consecutive time bins for the subsequent analyses.

Then, we summed up the integral of the oxy-Hb signals representing task-related activation (i.e., PSP versus rest and MSP versus rest) for the selected COIs included in each ROI, and these subjectwise activation values were finally entered into the second-level between-subject analyses, in which we tested whether the summated activation values for each ROI differed between the PSP and MSP tasks using paired *t* tests. IBM SPSS Statistics (ver. 22) was used for these analyses, and the level of significance was set at *P* < 0.05.

## 3. Results

All of the subjects were able to perform the tasks assigned in each experiment with ease. The mean number of pegs transferred within the 30-second PSP task blocks was 6.83, and the frequency of movements was approximately 0.23 Hz in the PSP and MSP tasks. We confirmed that none of the subjects missed any of the movements prompted by the metronome during the MSP task. So, behavioral activity was completely controlled between the two tasks because the MSP was based on the pace at which each subject performed the PSP task.

In the present study, 22 healthy subjects performed both tasks, and fNIRS measurements were obtained via 42 channels in each task, which resulted in 1848 fNIRS time-series datasets. We excluded 4 of the 1848 datasets (0.22%) due to considerable motion artifacts.

### 3.1. Activated Channels

The numbers of positively activated channels in each ROI (i.e., the number of channels selected through the within-subject channel-by-channel GLMs) demonstrated that there was no difference in the number of selected channels between the two tasks in any ROI (Table 1). This suggests that all of the prefrontal ROIs were positively activated compared with the baseline rest during both tasks.

**Figure 4:**
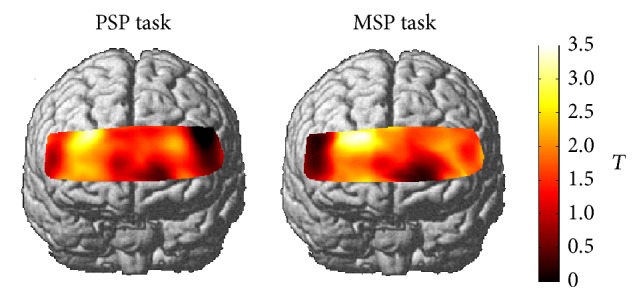
Group analyses of the PSP and MSP tasks. The differences in cortical activation between each task and the baseline rest were shown as* t* maps of the oxy-Hb signals.

Group analyses of both tasks were described as in Figure 4.

### 3.2. The Integral of the Oxy-Hb Signals

In the second-level between-subject analyses, we found that the FPPFC was activated to a greater extent during the PSP task than during the MSP task [*t*(21) = 1.769, *P* = 0.046]. No significant task-related differences (PSP versus MSP) were observed in other regions [right DLPFC:* t*(21) = 0.593, *P* = 0.280; left DLPFC:* t*(21) = −1.411, *P* = 0.086; right VLPFC:* t*(21) = 0.564, *P* = 0.289; left VLPFC:* t*(21) = −0.216,* P* = 0.415].

## 4. Discussion

The purpose of the present study is to compare the activation of the prefrontal cortex induced when the subjects performed a pegboard task at their preferred slow pace (PSP, the self-initiated condition) with that induced when they performed the same task at metronome slow pace (MSP, the externally triggered condition) using fNIRS. All of the prefrontal ROIs were activated compared with the baseline (resting conditions) in both tasks. However, we detected greater FPPFC (BA 10) activation (the integral of the oxy-Hb signals) during the PSP task than during the MSP task.

### 4.1. Evaluation of Self-Generated PSP

By counting the numbers of activated channels (compared with the resting conditions), we confirmed that all of the prefrontal ROIs, including the DLPFC (BA 9 and 46) and VLPFC (BA 44, 45, and 47), were activated during both tasks in our study. Previous PET studies demonstrated that the DLPFC (BA 9, 46, and 10) was activated during self-initiated movements and indicated that the DLPFC holds information about preceding interresponse intervals in working memory [8, 10]. In addition, the VLPFC plays a critical role in spatial working memory [30]. Working memory is assumed to be necessary for a wide range of complex cognitive activities [31]. In the present study, the subjects were required to retain information about the timing of the previous response in their working memory during both tasks, which resulted in activation of the DLPFC and VLPFC.

Comparing the activation induced by each task, we observed greater FPPFC activation during the PSP task than during the MSP task, which corresponded with the findings of previous PET studies involving similar tasks in which the FPPFC was examined [8, 10]. During the PSP task, the subjects were making a move while receiving feedback from their own input systems (e.g., their visual processing, auditory processing, motor effector systems, etc.). In the model of frontal hierarchy, the FPPFC is at the top of the hierarchy, and the DLPFC is located at a lower level [32, 33]. The DLPFC activation is involved in the monitoring and manipulation of externally generated information. On the other hand, the FPPFC is specifically involved in evaluating self-generated responses or plans for action [32, 34]. In addition, the FPPFC is known to play a role in coordinating and integrating the DLPFC and VLPFC. Fletcher and Henson suggested that the FPPFC works to maximize the function of the lateral prefrontal cortex (LPFC), which is concerned with maintenance, manipulation, and monitoring during task performance [35]. Taken together, the finding of the FPPFC activation during the PSP task suggests that the subjects evaluated and managed the internally generated PSP by coordinating the activity of other lower level prefrontal regions.

### 4.2. Free Decisions

We consider that the main difference between the PSP and MSP tasks was whether they involved timing decisions, which are required for self-initiated movements [8, 10]. Recent research has suggested that the FPPFC is a candidate for the region responsible for unconscious generation of “free decisions” about when tasks should be performed [36–38]. Thus, free decision processes, the neurological basis for which is the FPPFC activation, are suggested to have been responsible for the self-initiated pace set during the PSP in the present study. However, this remains speculative and needs to be addressed in future studies.

### 4.3. Technical Issues Associated with the fNIRS System

The differences between the findings of the present study and those of previous PET studies that detected activation of both the FPPFC and DLPFC during self-initiated tasks [8, 10] might have resulted from technical issues associated with fNIRS system used in our study. One of the problems with fNIRS is that the differential pathlength factor (DPF) varies among brain regions [39–41]. In the forehead, the DPF is roughly constant in the central region [39], whereas it decreases markedly in the frontotemporal region due to the presence of muscle [39, 41]. As the FPPFC is relatively free from such structural complexity, the task-dependent absorption changes in the FPPFC might have been measured more accurately than those in the LPFC by fNIRS in the present study.

### 4.4. Implications and Future Directions

In order to compare activation of the prefrontal cortex during the PSP and MSP task in the present study, we selected slow pace as the speed at which these tasks were performed. Slow pace can be easily synchronized with an external cue because it allows adequate time to prepare for the next cue. There are several reports that prefrontal cortex is independent of the frequency/speed of movement if the subjects maintain their constant pace [16, 42]. However, in a future study, we will compare preferred pace and metronome pace at another speed of pace.

Occupational therapy is a client-centered health profession that is concerned with promoting health and wellbeing through occupations. The primary goal of occupational therapy is to enable people to participate in the activities of everyday life [43]. The largest numbers of patients that currently require care in Japan are those with cerebrovascular diseases [44]. Cerebrovascular diseases most frequently affect brain function in adult patients [45]. However, the method to select the optimal pace for the patient has not been examined. In the present study, we demonstrated that activation of the prefrontal cortex was significantly greater during the PSP task than during the MSP task in an environment that reflected everyday life. These results will contribute to the development of rehabilitation approaches for patients.

## 5. Conclusions

Individuals have a preferred pace at which they perform voluntary repetitive movements. We confirmed that all of the prefrontal regions of interest were activated by both tasks. Among these prefrontal regions, we found that activation in the FPPFC (BA10) was higher during the PSP task than during the MSP task. The finding of the FPPFC involvement during the PSP task suggests that each subject evaluated and managed their internally generated PSP by coordinating the activity of other lower level prefrontal regions.

## Figures and Tables

**Table 1:** Comparison of the numbers of channels that were significantly activated by the PSP and MSP tasks.

ROI	No. of selected channels (mean (SD) for all subjects)		*t* value (df = 21)	*P* value
	PSP versus rest	MSP versus rest		
FPPFC	1.64 (1.29)	1.68 (1.39)	−0.18	0.86
Right DLPFC	2.36 (1.59)	2.09 (1.38)	1.24	0.23
Left DLPFC	1.55 (1.41)	1.82 (1.53)	−1.14	0.27
Right VLPFC	2.55 (1.74)	2.50 (1.47)	0.15	0.89
Left VLPFC	2.55 (1.63)	2.68 (1.67)	−0.45	0.66

Paired *t* test (*P* < 0.05).
